# The Current Applications of Metabolomics in Understanding Endometriosis: A Systematic Review

**DOI:** 10.3390/metabo15010050

**Published:** 2025-01-14

**Authors:** Blake Collie, Jacopo Troisi, Martina Lombardi, Steven Symes, Sean Richards

**Affiliations:** 1 Department of Biology, University of Tennessee at Chattanooga, Chattanooga, TN 37403, USA; 2 Department of Medicine, Surgery and Dentistry, “Scuola Medica Salernitana”, University of Salerno, 84081 Baronissi, Italy; 3 Theoreo Srl., Via Degli Ulivi 3, 84090 Montecorvino Pugliano, Italy; 4 European Institute of Metabolomics (EIM) Foundation, Via G. Puccini, 3, 84081 Baronissi, Italy; 5 Department of Chemistry and Physics, University of Tennessee at Chattanooga, Chattanooga, TN 37403, USA; 6 Section on Maternal-Fetal Medicine, Department of Obstetrics and Gynecology, University of Tennessee College of Medicine, Chattanooga, TN 37403, USA; 7 Department of Biology, Geology and Environmental Sciences, University of Tennessee at Chattanooga, Chattanooga, TN 37403, USA

**Keywords:** endometriosis, metabolomics, lipidomics, biomarker

## Abstract

Endometriosis is a common gynecological disease that affects approximately 10–15% of reproductive-aged women worldwide. This debilitating disease has a negative impact on the quality of life of those affected. Despite this condition being very common, the pathogenesis is not well understood. Metabolomics is the study of the array of low-weight metabolites in a given sample. This emerging field of omics-based science has proved to be effective at furthering the understanding of endometriosis. In this systematic review, we seek to provide an overview of the application of metabolomics in endometriosis. We highlight the use of metabolomics in locating biomarkers for identification, understanding treatment mechanisms and symptoms, and relating external factors to endometriosis. The literature search took place in the Web of Science, Pubmed, and Google Scholar based on the keywords “metabolomics” AND “endometriosis” or “metabolome” AND “endometriosis”. We found 58 articles from 2012 to 2024 that met our search criteria. Significant alterations of lipids, amino acids, as well as other compounds were present in human and animal models. Discrepancies among studies of significantly altered metabolites make it difficult to make general conclusions on the metabolic signature of endometriosis. However, several individual metabolites were elevated in multiple studies of women with endometriosis; these include 3-hydroxybutyrate, lactate, phosphatidic acids, succinate, pyruvate, tetradecenoylcarnitine, hypoxanthine, and xanthine. Accordingly, L-isoleucine and citrate were reduced in multiple studies of women with endometriosis. Including larger cohorts, standardizing testing methods, and studying the individual phenotypes of endometriosis may lead to more separable results.

## 1. Introduction

Endometriosis (EM) is a common gynecological disease that is classically defined as the presence of endometrial-like tissue outside of the uterus [1]. It affects approximately 10–15% of reproductive-aged women globally [2]. EM can be presented with superficial peritoneal lesions, ovarian cysts, or deep infiltrating scarring and adhesions that exceed a depth of 5 mm [1]. The pathogenesis of this condition is not yet fully understood. However, a few theories attempt to explain the cause of endometriosis. The theory of retrograde menstruation posits that the menstrual uterine contents are sloughed into the peritoneal cavity [3]. Other theories that focus on non-uterine origins include the coelomic metaplasia theory as well as the theory of benign metastasis [4]. It must be noted that the persistence of EM, on the other hand, must be due to a combination of hormonal, inflammatory, and immunologic factors [2].

EM is characterized by a large symptomatic profile. The range of EM-associated symptoms includes chronic pelvic pain, dysmenorrhea, dyspareunia, dysuria, dyschezia, metrorrhagia, diarrhea, constipation, infertility, and myofascial pain [5]. It has been documented that 35–50% of women experiencing pelvic pain, infertility, or both suffer from EM [4]. This debilitating condition may also lead to a decrease in the quality of life in a large portion of patients [6]. The American Society for Reproductive Medicine (ASRM) has established a classification of EM based on the number, size, appearance, and location of peritoneal and ovarian lesions. According to the ASRM guidelines, EM can be classified into stages I, II, III, and IV, which correspond to minimal, mild, moderate, and severe [7].

The diagnosis of EM is rather difficult. Currently, the gold standard relies on invasive laparoscopic observation [8]. There is a reported average delay of 6.7 years between the onset of symptoms and the diagnosis [9]. The lack of a unique symptom profile as well as there being no correlation between the stage of EM and the presence of symptoms are the main factors contributing to this gap [10]. The invasiveness and costs associated with a laparoscopic procedure also make diagnosis a challenge. A non-invasive or minimally invasive biomarker of endometriosis may help improve diagnosis time. Currently, no biomarkers for the diagnosis of EM have been viable for use in a clinical setting [10].

Treatment options for EM are also currently very limited. The current options for treatment include hormonal therapy, pain management, and surgery [5]. Some of the surgery techniques include ablation of uterosacral nerves by employment of endocoagulation, hysterectomy with bilateral salpingo-oophorectomy, excision or removal of endometrial implants, presacral neurectomy, and electrocautery or laser treatment [2]. Surgical options are shown to have limited success with a recurrence rate of symptoms in 40–50% of patients in 5 years [11]. A biomarker that can detect EM in earlier stages may lead to greater surgical outcomes with less recurrence.

Metabolomics is the study of the identities and presentation of the array of low-molecular-weight organic and inorganic metabolites of biochemical processes to reveal the phenotype of a cell, tissue, and organ [12] (p. 16). It has been used in the discovery of biomarkers in diseased tissue, to assess the efficacy of pharmaceuticals and to determine the pathophysiology of diseases [12] (p. 17). Ultimately, metabolomics has proved to be useful in biomedical applications. The two approaches in metabolome analysis are targeted and untargeted. Targeted metabolomics refers to analyzing a subset of metabolites, while untargeted metabolomics refers to the broad analysis of metabolites within a sample [13] (pp. 30–31). Common samples selected for metabolomic analysis include biofluids (e.g., blood, plasma, serum, and urine) and tissue samples.

Analytical platforms commonly used for the determination of metabolomic profiles typically include either NMR or chromatography (gas or liquid) paired with mass spectrometry. Some advantages of NMR include its nondestructive quality, reproducibility, and ease of sample preparation, whereas the high sensitivity of mass spectrometry and high chromatographic resolution of GC and LC makes them invaluable tools in metabolomics [14]. These techniques have high-throughput capability and generate large datasets often resulting from the detection of hundreds of metabolites among hundreds of samples. Data analysis often relies on multivariate statistical comparisons to produce an applicable biological insight. Some of the most widely used tools for data analysis in metabolomics include the Partial Least Square regression and Principal Component Analysis [15] (pp. 291, 333).

The purpose of this review is to provide an insightful overview of the current applications of metabolomics in EM. Two recent review articles have summarized metabolomic biomarkers in EM identification [16,17]. Both focus on metabolite alterations among human studies for potential use in disease identification. However, the present review goes beyond those reviews and highlights the most promising biomarkers for diagnosis as well as discrepancies within the relevant literature. Additionally, studies with animal models that report metabolite alterations in EM are also reviewed. Finally, the present review highlights how metabolomics is applied to understanding treatment mechanisms, symptoms, and the relation of external factors to EM.

Currently, targeted and untargeted metabolomic approaches are used as tools to discover biomarkers for diagnosis, to understand the pathophysiology, and to describe how external factors alter the metabolome of EM patients. Within the current literature, case-control studies, pilot studies, and interventional studies have been conducted across blood plasma, blood serum, follicular fluid, peritoneal fluid, endometrial tissue samples, endometrial fluid, cumulus cells, various cell types, as well as animal models. Promising discoveries of minimally invasive and non-invasive biomarkers were presented. Interventional studies have aimed to explore how traditional Chinese medicine can affect the metabolome in mouse models when treated for endometriosis. Alterations in the EM metabolome due to diet, pollutants, and the microbiome are described. Lastly, this review aims to offer future directions for the field of metabolomics and address current limitations.

## 2. Materials and Methods

### 2.1. Search Strategy

This review followed the PRISMA (Preferred Reporting Items for Systematic Review and Meta-Analysis) guidelines [18]. We used the PRISMA checklist when writing our report [19]. In May 2024, a systematic literature search was conducted using the PubMed and Web of Science databases, applying the following search queries: “metabolomics” AND “endometriosis” or “metabolome” AND “endometriosis”. Additional articles were identified through a manual search of reference lists and on Google Scholar. Eligible manuscripts were peer-reviewed, published as either a journal article or conference paper, and must have been final print material (pre-prints were not eligible). Manuscripts were not restricted to any date range; however, the resultant date range of eligible papers was from 2012 to 2024. Two people worked independently to collect manuscripts.

### 2.2. Inclusion Criteria

We included original research articles that applied metabolomics to studying the pathogenesis, treatment, and diagnosis of EM. Studies conducted in vitro and in vivo were selected. We also chose to include metabolomic studies of EM in both human and animal models.

### 2.3. Exclusion Criteria

Review articles were excluded from the results. Any articles with abstracts that did not meet the search criteria were excluded. Methodical studies on the design of metabolomic testing in EM patients were also excluded. Studies that were determined to contain selection bias, performance bias, detection bias, or reporting bias were excluded. Conference abstracts, books, graduate degree theses, and other non-peer-reviewed publications were also excluded.

### 2.4. Data Management and Extraction

**Figure 1:**
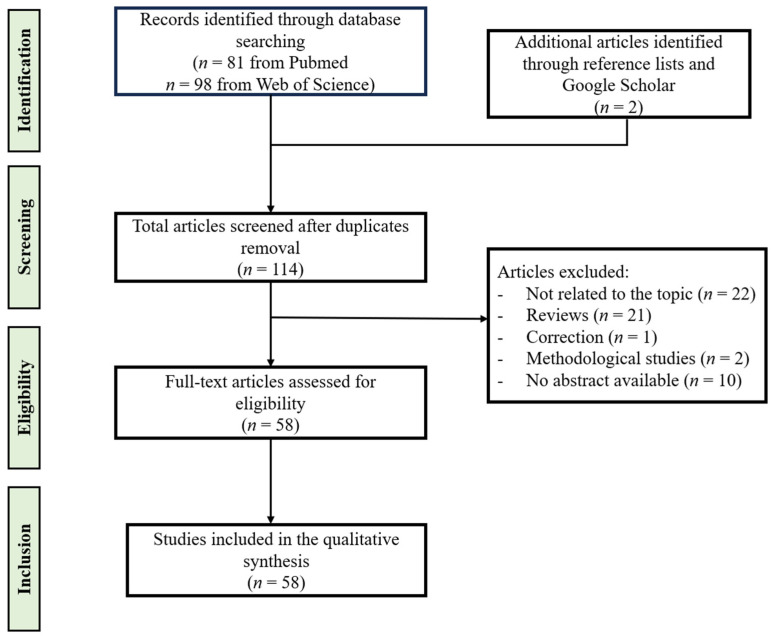
Flow diagram of the study selection process.

The reference manager Zotero was used to organize data. Articles that met the inclusion criteria were loaded into Zotero. From the selected articles, we chose to extract information regarding (1) the type of study, (2) the location of the study, (3) the number of test subjects, (4) the sample source, (5) the limitations, (6) the analytical method, (7) and the identities of significantly altered metabolites between cases and controls. Extracted information was organized and used to develop the review. The significantly altered metabolites in both human and animal models that were found to distinguish an EM group from controls were organized into data tables based on their sample source. Figure 1 summarizes the study selection process.

## 3. Results

### 3.1. Search Results

The initial results from Pubmed and the Web of Science yielded 81 and 98 results, respectively. From the 179 results, 67 duplicates were removed to leave a total of 112 results. After screening the results, 22 did not meet the search criteria, 21 were review articles, 10 had no abstract, 1 was a correction, and 2 were methodical studies. After removing these results, 56 articles that met the search criteria remained. Two additional articles were found by scanning the sources of the selected articles. These articles were then located in Google Scholar and added to the pool of articles. A total of 58 articles were selected for use in this review. The final pool of articles ranged from the year 2012 to 2024. Of the final results, 12 of these articles were animal studies, 3 were conducted in both animals and humans, and 43 were conducted in human models. The articles contained case-control studies, interventional studies, pilot studies, cross-sectional studies, and prospective observational translational studies. Metabolomic studies were carried out on samples of serum, plasma, follicular fluid, peritoneal fluid, tissue samples, cell cultures, endometrial fluid, and urine in human models. The animal studies were conducted in peritoneal lavage fluid, tissue samples, feces, and serum. Different stages of EM as well as certain phenotypes of this condition were addressed in the research.

### 3.2. Biomarkers in Human Studies

The human studies took place across the countries of India, Iran, Slovenia, Brazil, Italy, Spain, China, Russia, Singapore, Granada, Belgium, France, the United Kingdom, Turkey, and the United States. A total of thirty-four case-control studies, three cross-sectional studies, two pilot studies, four prospective observational studies, and one interventional study were found. The sample sizes ranged from 16 to 180 samples. Of the studies that documented the stages of EM, five focused on minimal or mild stages (I, II), one included mild and moderate stages (II, III), thirteen studied moderate or severe stages (III, IV), and twelve included samples from three or more stages of EM. In this section, we address the alterations of amino acids, lipids, and other compounds that were found to distinguish an endometriosis group from a control group in human models. Table 1 summarizes the significant metabolite differences of samples from humans with EM relative to those without EM.

#### 3.2.1. Amino Acids

Studies conducted in human models were able to differentiate patients with EM from a control group based on their specific metabolomic signature. One of the most notable classes of metabolites studied was amino acids. Significantly altered levels of alanine, histidine, arginine, asparagine, glutamine, glutamate, isoleucine, leucine, lysine, threonine, valine, methionine, phenylalanine, proline, serine, tyrosine, and tryptophan were detected.

Isoleucine was the only amino acid found to be consistently lower in patients with EM. Isoleucine was found to be downregulated in both plasma [22,23,24] and follicular fluid [31]. This is the only amino acid to not have discrepancies across multiple studies and different sample types.

The present search found many discrepancies among the levels of detected amino acids between studies. For those amino acids found in more than one study and with discrepancies between those studies, potential reasons for the discrepancies were explored and noted below. For example, a Chinese study on eutopic endometrial samples from 29 women with EM and 37 infertile women found both leucine and lysine to be upregulated in the EM group [37]. In contrast, an Indian study also on eutopic endometrial tissue samples from 95 women with EM and 24 controls found leucine and lysine to be decreased [36]. The contradicting results may be due to the small sample sizes present in both studies. Both of these studies used different identification techniques, as Li et al. [37] used ultra-high-performance liquid chromatography coupled with electrospray ionization high-resolution mass spectrometry and Dutta et al. [36] used NMR. Tissue samples were also taken at different phases of the menstrual cycle, as Li et al.’s [37] was taken during the follicular phase and Dutta et al.’s [36] was taken during the luteal phase. Leucine was noted to be upregulated in only one more study [24], while it was downregulated in four more studies [22,23,31,35]. Dutta et al. [24] may have received opposite results in serum because those samples were collected during the secretory phase, whereas Jana et al. [23] collected samples during the early follicular phase, and Maignien et al. [22] included women at different stages of the menstrual cycle. It must also be noted that Maignien et al. [22] included women undergoing hormonal treatment, unlike Dutta et al. [24], who only included serum samples from women who had not received hormonal treatment three months prior to sample collection.

Alanine was found to have contradicting results in stage I and stage II EM. An Indian study on blood serum from 22 EM patients and 23 controls found alanine to be upregulated in EM patients [24]. However, a separate study discovered that alanine was downregulated in the eutopic endometrial tissue of EM patients [36]. The inverse relationship between these results may be due to the different sample types used. Alanine was also found to be downregulated in four studies: two in follicular fluid [30,35] and two in serum [23,28]. Angioni et al. [28], who disagreed with the direction of regulation of alanine in the study of Dutta et al. [24], studied an EM group consisting of 22 patients who all had deep infiltrating endometriosis. It must be also noted that Angioni et al. [28] found a significant difference between the median ages of their EM and no-EM group. The difference in the stages of EM may explain the reported difference in alanine levels.

Histidine was found to be significantly altered in three studies [20,22,24]. Despite Wei et al. [20] analyzing stage III and IV follicular fluid and Dutta et al. [24] analyzing stage I and II blood serum, both studies found histidine to be significantly increased. Conversely, a study on blood plasma conducted in France that had 46 EM patients and 21 controls found histidine to be decreased [22]. These differences may be because the EM group in the study of Maignien et al. [22] contained 23 with endometriomas and 23 with deep ovarian EM, while the other two studies did not report the phenotypes of endometriosis. Another possible explanation is that 21 of the EM samples and 8 of the control samples were taken from women who were under hormonal treatment, which could have altered the metabolomic signature of both the EM and control groups [22]. Lastly, the use of exogenous gonadotropins during follicular fluid collection could have altered the metabolome in the study of Wei et al. [20] and caused it to have results in contradiction with Maignien et al. [22].

Arginine was lowered in both the serum [23,24] and follicular fluid [31], while it was upregulated in both the plasma [40] and eutopic endometrial tissue [37]. The study conducted in plasma contained 23 healthy controls and 50 endometriosis patients with samples taken during the follicular and luteal phase [40]. It must be noted that this study was unable to find statistically significant metabolite differences until the profiles of the EM and control group were studied according to their menstrual cycle phase [40]. There was only enough samples present to study the follicular phase in this study [40].

Asparagine was significantly decreased in the serum of 75 EM compared to 60 controls [23], while it was increased in the eutopic endometrial tissue [37]. As noted with alanine and arginine, there was an inverse relationship between the levels of asparagine in the serum and endometrial tissue.

Glutamine was upregulated in both serum samples with severe-stage EM [29] and in the follicular fluid of patients with moderate- and severe-stage EM [20]. On the contrary, glutamine levels were decreased in the serum of two studies [22,44], one of which was an Indian study with 39 endometriosis patients and 48 controls [44]. A possible explanation for Murgia et al. [29] having opposite results to the other studies is that their study included the most severe form of this condition, all with deep infiltrating EM, and they had a very low sample size of 22 EM patients and 15 controls. Wei et al. [20], who had 25 EM patients and 25 infertile controls, found many significantly altered metabolites. It was found that 36 metabolites were significantly upregulated and 17 were downregulated [20]. Also, it must be noted that the use of exogenous gonadotropins could have altered the metabolic profile in this study [20].

Threonine was found to have contradicting results in both plasma and follicular fluid. It was upregulated in two studies [24,30] and downregulated in two studies [31,36]. It must be noted that the discrepancy among the results in the follicular fluid may be because Lazzarino et al. [31] studied EM in a pooled group with other infertility conditions such as polycystic ovary syndrome, reduced ovarian reserve, unexplained infertility, and genetic infertility, which could have led to an altered metabolome when compared to controls.

Valine was found to be significantly altered in nine studies. Four of them noted that valine was increased [24,27,40,42], while five found valine to be decreased [22,25,28,31,35]. As mentioned previously, one of the studies that noted the decrease in valine used a pool of infertility conditions rather than EM alone [31]. Similarly, another study also looked at various causes of infertility alongside eight endometriosis patients [25]. One study focused solely on ovarian endometriosis, unlike the rest of the studies that reported significant alterations in the levels of valine [42]. Small sample sizes were found across all the studies, as the highest number of total samples among the nine studies was 67.

Phenylalanine was increased in follicular fluid [20], while it was decreased in tissue samples [36]. This difference in phenylalanine levels may be accounted for by the differences in sample types and stages of EM.

Proline was found to be increased in all studies [20,28,36,44] except for one [35]. As with other discrepancies, the difference reported by Santonastaso et al. [35] may be related to their study having a very low sample size, with only 16 EM samples and 7 controls, which happened to be the lowest out of all five studies. It is possible that the effects of using external gonadotropins on the metabolome during the extraction of follicular fluid could have been amplified due to the small sample size present in this study. There is also a significant difference in the mean age between the EM and control groups in Santonastaso et al. [35] compared to Dutta et al. [36].

Tyrosine was reported to be decreased in the serum [22] and follicular fluid [30], while it was increased in tissue samples [37]. The most obvious reason for the difference in the reported changes in the metabolome may be due to the different sample types studied. Li et al. [37] studied stages I and II EM, while Pocate-Cheriet et al. [30] had a mean ASRM stage of 2.96, and Maignien et al. [22] studied stage IV EM. Lastly, Li et al. [37] used ultra-high-performance liquid chromatography coupled with electrospray ionization high-resolution mass spectrometry (UHPLC-ESI-HRMS), whereas the other two studies used ^1^H-NMR [22,30].

Tryptophan was reported to be downregulated in two studies [29,31] and upregulated in one study [20]. Aside from previously noted factors, the differences in ethnicities of the study participants (China and Italy) may play a role in this difference in the metabolome.

Despite the large number of contradictions present among the results, amino acids play an important role in physiology, are routinely detected, and may serve as biomarkers or drivers of EM metabolome signatures with more conclusive research.

#### 3.2.2. Lipids

Another highly studied class of metabolites is lipids and their derivatives. The literature has shown many significant changes in the lipidome of EM patients. While some studies identified specific lipids that were altered, others revealed the general class. A few studies noted alterations in the general classes of lipids [23,24,30,35] and unsaturated lipids [25]. For example, one study found lipids were decreased in the metabolome of EM [24] while they were increased in the rest of the studies [23,30,35]. The contrasting results may be because Dutta et al. [24] studied stages I and II of EM, while the two studies that found lipids to be increased studied more advanced stages of EM [30,35]. One study supports this claim by finding lipids to be more prevalent in stages III and IV EM than in stages I and II [35]. The altered lipids discussed in the reviewed literature included various sphingomyelins, phospholipids, carnitines, lipid derivatives, fatty acids, triacylglycerols, as well as steroid derivatives.

Relative triacylglycerol abundances also showed contrasting results in the literature. A study that contained 50 serum samples of EM patients and 50 control samples found triacylglycerols to be elevated in the EM group [32]. However, a study using endometrial fluid noted triacylglycerol levels to be decreased in the EM group [51]. The discrepancy may be caused by a difference in sample types and/or testing techniques since Braga et al. [32] used ESI-MS and Domínguez et al. [51] used UPLC-MS.

The most prevalent phospholipid class described in the literature is the phosphatidylcholines, which were found to be altered in six studies [20,33,39,49,50,51]. Glycerophosphatidylcholine was upregulated in the serum of EM patients in one study [24]. Different phosphatidylcholines were significantly altered in both the control and EM groups of two studies [50,51]. One was a Spanish study on endometrial fluid from 12 EM samples, and 23 controls found that phosphatidylcholine 22:6/0:0 was noted to be increased in EM patients, while phosphatidylcholine 42:6 was decreased [51]. The other article found that phosphatidylcholines were abundant in the control group, while different phosphatidylcholines were abundant in the EM group. Other phospholipids that were detected to be altered include phosphatidylinositol [47], phosphatidylinositol bisphosphate [50], phosphatidylglycerol phosphate [50], phosphoethanolamine [34], and phosphatidylserine [38,49,50]. Phosphatidylserine was found to be downregulated in two studies [49,50], while it was upregulated in one study [38]. It was noted that two studies conducted on follicular fluid samples from patients with stage III and IV EM found differing results on the presence of phosphatidylserine in EM patients [38,50]. The difference between these results may be because of the small sample sizes in both studies as well as the different types of mass spectroscopy used in the study. One of the studies used electrospray tandem mass spectrometry [50], whereas the other used ultra-performance liquid chromatography mass spectrometry [38].

Some derivatives of phospholipids that were altered are the lysophosphatidic acids [20,38], lysophosphatidylcholine [20,34,38,46], lysophosphatidylethanolamine [49], lysophosphatidylglycerol [38], and lysophosphatidylinositol [20,38,47]. Phosphatidic acid was upregulated in the follicular fluid of two studies [38,48], whereas Wei et al. [20] was the only study to find lysophosphatidylcholine to be downregulated. An interesting finding is that Cordeiro et al. [38] and Wei et al. [20] found opposite results for lysophosphatidic acids, lysophosphatidylcholine, and lysophosphatidylinositol despite both studies being conducted in the follicular fluid of stage III and stage IV infertile patients with EM. Both studies had low sample sizes, with Wei et al. [20] having 25 EM patients and 25 controls and Cordeiro et al. [38] having 18 EM patients and 22 controls. Another possible explanation for the difference between these studies is that Cordeiro et al. [38] had a higher mean age in both the control and the EM group than Wei et al. [20]. Lysophosphatidylinositol was upregulated only in Cordeiro et al. [38]. The difference between Cordeiro et al. [38] and Dai et al. [47] may possibly be due to the difference in the stages of EM studied. Cordeiro et al. [38] studied only stage III and IV EM but Dai et al. [47] studied all stages of EM.

Sphingomyelins were found to have different results among the different types of samples. A study conducted in Slovenia had 40 EM patients and 52 controls with moderate- to severe-stage EM and found that sphingomyelins were increased in the blood plasma of endometriosis patients [52]. While studies in endometrial fluid [51], peritoneal fluid [33], and cell cultures [41] found the opposite result for sphingomyelins. Some possible explanations for Vouk et al. [52] having different results than the other studies may be due to the following factors: the plasma samples were collected at different phases of the menstrual cycle; many of the controls and patients took medication during the prior week; and obese patients were in the control group but not in the EM group. A combination of these factors may have altered the metabolome profiles and led it to have differing results than the other three studies.

The carnitines that were found to be altered included acylcarnitines [20,33,39], lauroylcarnitine [45], hexadecenoylcarnitine [45], myristoylcarnitine [45], oleylcarnitine [45], and tetradecenoylcarnitine [39,45]. Lauroylcarnitine, myristoylcarnitine, hexadecenoylcarnitine, oleylcarnitine, and tetradecenoylcarnitine were all found to be increased in the plasma of a study that contained 25 women with EM and 19 controls [45]. Differences were reported in the alterations of acylcarnitines, as Wei et al. [20] and Vouk et al. [33] found acylcarnitines to be decreased, while Li et al. [34] found them to be increased. Although there is a disagreement in the acylcarnitine levels in peritoneal fluid, it must be noted that Li et al. [34] presented different specific acylcarnitines than Vouk et al. [33]. Aside from this, the opposing results may be because Vouk et al. [33] used only ovarian EM patients, whereas Li et al. [34] did not report its patients having ovarian EM.

Free fatty acids appeared to be altered in both serum [23] and follicular fluid [35,38]. One study reported increases in the levels of fatty acids; however, the other two studies reported decreases in fatty acids [38] in the EM group [23,35]. The method of metabolomic analysis used in this study was UPLC-MS, while the other two articles that were in agreement on the decrease in fatty acids used ^1^H-NMR.

One study noted that five steroid compounds were increased in serum including 2-methoxyestradiol, 2-methoxyestrone, dehydroepiandrosterone, aldosterone, and deoxycorticosterone [21]. Progesterone was detected to be significantly upregulated in the serum, follicular fluid, and peritoneal fluid of patients with ovarian endometriosis [53]. EM may play a role in altering the lipidome by increasing the presence of steroid compounds.

Despite some variations in the literature, EM has a significant effect on the lipidome. This indicates that the alterations of lipids may be part of the underlying mechanisms of EM.

#### 3.2.3. Other Compounds

Other compounds were also found to be altered in the metabolic signature of EM in human models. Some of the compounds are involved in energy production. Lactate, which is involved in anaerobic energy production, was found to have agreement in the literature. Lactate was upregulated in all seven of the reported studies [23,24,25,30,31,35,42]. Pyruvate, which is the product of glycolysis, was upregulated in serum samples [23] and in follicular fluid [30,42]. The uniform results in the literature indicate that these metabolites play an important role in the pathophysiology of EM.

3-hydroxybutyrate, a ketone body, was another one of the few compounds that showed uniform results across multiple studies. It was found that this ketone body was significantly upregulated in the serum and follicular fluid of patients with both mild and severe forms of EM [22,24,28,29,30]. A similar compound, 2-hydroxybutyrate, was found to be upregulated in serum [23,24] and decreased in follicular fluid [25].

Acetone was decreased in one study [24] and increased in two studies [22,30]. It must be noted that the difference among these studies may be because Dutta et al. [24] studied stage I and stage II EM, while Pocate-Cheriet et al. [30] studied stage IV, and Maignien et al. [22] had a mean ASRM stage of 2.96. This indicates that acetone may be decreased in early-stage EM and increased in late-stage EM.

Glucose was found to be significantly altered among various studies of different sample types [23,24,25,30,31,35,42]; however, there were some disagreements in the levels present. Only two of the presented studies indicated an upregulation of glucose in EM [25,42], while the rest of the studies indicated a decrease in glucose [23,24,30,31,35]. Both studies that presented disagreements with the majority of the research contained very small sample sizes. One contained only 8 EM follicular fluid samples [25] and the other contained only 12 EM samples [42]. Other glucose compounds that were noted to be significantly increased in follicular fluid are β-D-glucose 6-phosphate and glucose-1-phosphate [20].

Succinate, a tricarboxylic acid cycle (TCA) intermediate, was increased in serum in two studies [23,24] as well as in peritoneal fluid in one study [26]. The presented studies indicate that the increase in succinate is present in both the early stages of EM [24,26] as well as late stages of EM [26]. However, it is found that severe stages of EM accumulate higher levels of succinate than in the minimal or mild stages of EM. However, another TCA intermediate, citrate, was found to be decreased in two studies [25,30]. Both studies used H-NMR to analyze the metabolomic profiles of follicular fluid from patients with late-stage EM.

The nucleobases that appear to be altered are uracil [31], thymidine [20], guanosine [37], and cytosine [31].

Xanthine, which is a purine metabolite, was increased in two studies in follicular fluid [20,31]. Aside from the studies using the same sample type, these studies also both used HPLC-MS for their method of metabolite detection [20,31]. Other purine metabolites, including inosine, xanthosine, and hypoxanthine, were increased in eutopic endometrial samples [37], whereas uric acid was significantly decreased in eutopic endometrial tissue [37].

Creatine, an amino acid derivative, was significantly altered in three studies [23,24,30]. This metabolite was decreased in follicular fluid [30], while contradictory results were found in serum [23,24]. As mentioned above, serum samples from these two studies were taken from different phases of the menstrual cycle, which may have caused there to be different expressions of this metabolite within each cohort.

The three dipeptides, glutamyl arginine [20], glutamyl phenylalanine [20], and phenylalanyl-isoleucine [39] were significantly increased in the endometriosis group. It was also found that five indole metabolites were shown to be upregulated in follicular fluid from patients with stage III and IV EM. These metabolites are indole, dihydroindole, indole lactic acid, indole-3-acetamide, and 5-hydroxyindole acetic acid [20]. Despite some of the present discrepancies, some of these metabolites indicate a high demand for energy in endometriosis.

### 3.3. Animal Models

Case-control studies have been conducted in animal models of non-human primates and mice. There are far fewer case-control studies conducted in animal models compared to humans. A total of seven studies were found that presented significant differences between an EM group and a control group. Table 2 highlights the significantly altered metabolites found in various animal models. The studies were conducted in samples of serum, tissue, peritoneal lavage fluid, colonic flushes, and feces. The reported animal model results generally agree with research conducted using human models.

A Chinese study conducted untargeted metabolomics in the follicular fluid of both human and mouse models with 6 EM mice and 5 controls found that both lysophosphatidylcholine and phosphoethanolamine were upregulated in both models [34].

Dutta et al. [59] found alterations in the serum metabolome among 10 EM donor mice, 10 EM recipient mice, eight macrophage-induced inflammation controls, and eight healthy control mice. This study determined that lysophosphatidylcholines were also upregulated [59]. Other compounds such as lysophosphatidylethanolamine, phosphatidylcholines, phosphatidylethanolamine, plasmeny-phosphatidylethanolamine, sphingomyelins, and triacylglycerols were detected to be altered [59].

Atkins et al. [57] studied alterations in the metabolome in a non-human primate model with the species *Macaca fascicularis* and *M. mulatta*. This study also addressed mitochondrial function in the endometrial tissue and contained 17 non-human primates with EM and 8 controls. The significant metabolites that were found to be lowered were carnitine, FAD, NADH, creatine phosphate, malic acid, and tryptophan [57]. The finding of lowered tryptophan levels agrees with two human studies [29,31].

Four studies also investigated the associations of microbiota on the metabolome of mice with EM [54,55,56,58]. Ni et al. [56] found that chenodeoxycholic and ursodeoxycholic acids were increased, while alpha-linolenic acid and 12,13s-epoxy-9z,11,15z-octadecatrienoic acid were decreased in the feces of C57BL/6J mice with EM. Chadchan et al. [58] used antibiotics to deplete the microbiota in mice with EM and no EM. It was found that n-butyrate, valerate, and iso-butyrate were lowered in the EM groups [58]. Chadchan et al. [54] followed a similar approach of using antibiotic-induced microbiota-depleted mice, while instead looking at the causal role of microbiota in EM. It was found that 2-aminoheptanoic acid, lactic acid, maltose, n-acetylaspartic acid, and quinic acid were increased in the EM mice [54]. One last study assessed the association of gut dysbiosis and immune response in 6–8 week-old BALB/c mice [55]. The mice were studied in four groups: ovariectomized mice treated with estradiol, ovariectomized mice with transplanted EM tissue and treated with estradiol, a control of non-ovariectomized mice with no transplanted endometriosis tissue, and non-ovariectomized mice with transplanted endometriosis tissue [55]. Metabolomics analysis of colonic flushes found decreased levels of acetic acid, propionic acid, butyric acid, and valeric acid when comparing the ovariectomized EM group to the control group of mice [55]. It was also noted that succinate, malate, glutamate, fumarate, phosphoenolpyruvate, glucose-6-phosphate, fructose-6-phosphate, 3-phosphoglycerate, and 2-phosphoglycerate were upregulated in the serum of non-ovariectomized EM mice relative to control mice [55]. Overall, animal models play a valuable role in understanding the underlying mechanisms of EM.

### 3.4. Correlation of the Endometriosis with External Factors

Metabolomics has also been applied to understand the correlation of outside factors with EM. A study sought to determine how adherence to the Mediterranean diet is associated with infertility caused by EM and recurrent implantation failure [60]. It was found that women with EM had lower levels of polyunsaturated fatty acids [60]. These compounds play a role in maintaining reproductive health and were found to be higher in women with high adherence to the Mediterranean diet [60].

Other studies used metabolomics to investigate the association of microbiota-derived metabolites with EM in mouse models. It was found that microbiota-derived quinic acid promotes EM [54]. Conversely, microbiota that produce short-chain fatty acids and n-butyrate were found to protect against the progression of EM [58]. Furthermore, another study found a decrease in butyric acid in non-ovariectomized EM mice [55]. An abundance of *Ruminococcus gnavus*, a short-chain fatty acid producer, may prevent inflammation related to EM [55]. Lastly, a study concluded that *Tuzzerella* and glutamine were significantly lower in tissue from patients with ovarian endometriosis compared to controls [61]. The decrease in glutamine may be related to the alteration of the microbiome in EM. It was hypothesized that glutamine may also play a role in reducing chemotactic factors of Treg cells, which have been associated with immune suppression [61].

The association between persistent organic pollutants and EM as well as their effect on the metabolome was assessed in two studies [62,63]. One found that polychlorinated biphenyls were positively associated with EM and that downregulated bile acid and lipase activity were present in EM patients [62]. The other study discovered that the persistent organic pollutant that was most correlated with deep endometriosis was trans-nonachlor [63]. Exposure to this pollutant was correlated with an increase in 2-hydroxybutyrate [63]. The application of metabolomics to external factors such as pollutants may help elucidate the causes of EM.

### 3.5. Symptoms and Treatments

Pain is experienced by many patients with EM. A study conducted on the epithelial cells of EM lesions revealed that these cells were able to produce the metabolite related to neuropathic pain including epinephrine, normetanephrine, 7,8-dihydroneopterin, and 7,8-dihydrobiopterin [64]. Symptom progression of EM is not fully understood. Metabolomics can provide new insights into symptoms such as pain in EM by isolating potentially dysregulated biological pathways. This not only provides a more detailed understanding of the condition but it also can help guide the development of new treatment options. Surgery is an option for the treatment of pain. However, the success of surgery in alleviating pain is not always guaranteed. A study investigated metabolites in pre-surgical blood samples of early-stage EM for pain associated with endometriosis post-surgery [65]. Lysophosphatidylethanolamine and lysophosphatidylcholines were associated with an increased risk of post-surgical pain, whereas pregnenolone sulfate and fucose indicated a decreased risk of post-surgical pain [65]. Although the previous results need to be validified, they help provide a better understanding of the recurrence of EM. Prognostic biomarkers will allow for a more targeted treatment of EM.

Metabolomics has also been used to determine how the treatment of EM affects the metabolome in humans. A study in follicular fluid of women with severe-stage ovarian endometriosis sought to determine the effects of progestin-primed ovary stimulation (PPOS) on the metabolic signature [66]. It was found that PPOS significantly increased amino acids, such as proline, arginine, and threonine, which may be vital for oocyte development [66]. Indeed, this study indicates that biomarker profiling can help advance assisted reproductive technologies, which may help improve infertility in patients with EM with further research.

A few studies also sought to understand the mode of action of Chinese traditional medicines through metabolomic profiling. The Chinese medicine studied included Protoberberine [67], Xiaoyi Yusi decoction [68], Guizhi Fuling Wan [69], paeoniflorin [70], Gui-Zhi-Fu-Ling-Capsules [71], paeonol [72], Resveratrol [73], and Sparganium stoloniferum-Curcuma phaeocaulis [74]. Protoberberine [67], paeonol [72], and Resveratrol [73] reported efficacy in animal models, and Xiaoyi Yusi decoction improved kidney deficiency in EM patients [68]. Liu et al. [72] applied metabolomics to understand the drug mechanism of paeonol in treating EM. Sparganium stoloniferum-Curcuma phaeocaulis was able to inhibit the growth of EM in a rat model by restoring the gut microbiota and altering the metabolism [74]. All the studies were able to detect changes in the metabolomic profiles of the treatment groups as compared to the controls. For example, it was found that Resveratrol was able to target the altered lipidome of EM patients by increasing sphingolipids and decreasing glycerolipids and phospholipids [73]. Metabolomics provides insight into drug mechanisms that may lead to more effective treatment of EM.

## 4. Discussion

### 4.1. Biochemical Pathway Alterations and Metabolite Significance

Many significant alterations in amino acids, lipids, and other compounds were found in the literature. The ability to distinguish specific alterations shows that EM has a unique metabolic fingerprint. A similar condition to EM is adenomyosis. Adenomyosis is characterized by stroma and endometrial glands within the uterus rather than growth outside of the uterus [75]. One article that investigated the metabolic profile of adenomyosis found that levels of 3-hydroxybutyrate in serum were higher in the adenomyosis group [75]. Relative to the research in EM, this metabolite was found to be lower in serum. Despite the similarity in these conditions, EM is shown to have a unique metabolic profile.

As mentioned in the introduction, there is a big need for a non-invasive or minimally invasive diagnostic test for EM. The development of a blood or urine-based test would prevent a large delay in the treatment of EM. Metabolomics shows great potential in producing specific metabolites for such a test. Although many studies recognized altered metabolites, the results of these studies must be clinically validated before the test can be used diagnostically.

**Figure 2:**
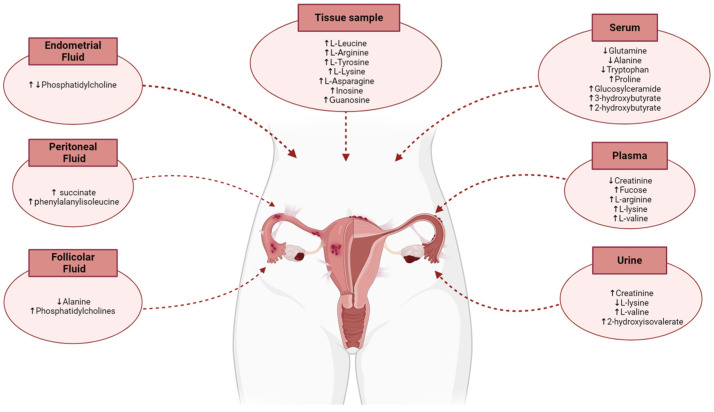
Graphical representation of metabolomic alterations identified in various reproductive tissues and body fluids from human studies. Arrows indicate specific increases or decreases in metabolite levels.

Based on these individual metabolites, conclusions can be drawn about the affected biochemical pathways in EM conditions. Figure 2 provides a summary of notable metabolomic alterations observed in human studies, specifically focusing on key metabolites across various bodily fluids and tissues associated with the reproductive system, reflecting the complexity and specificity of metabolic shifts in each area.

Some common explanations for the alterations of amino acids are elevated TCA intermediates, increased need for gluconeogenesis, and the upregulation for growth as in malignant conditions such as cancer [23,40,44]. Glutamine specifically can feed into the TCA cycle to replenish intermediates [44]. Serine and methionine may be used to increase pyruvate levels for the TCA cycle [76,77]. Lower levels of serine and methionine may indicate these amino acids may be utilized to increase the TCA cycle. The reported higher levels of threonine may indicate that the TCA cycle is disrupted as they may not be able to enter the TCA cycle [30]. Gluconeogenic amino acids, such as aspartate and alanine, feed into gluconeogenesis to produce glucose due to high energy demands, which could explain why they are altered [23]. Other gluconeogenic amino acids such as glutamine, glutamate, valine, isoleucine, threonine, and histidine were found to be reduced in serum, which may indicate increased gluconeogenesis [22]. Alanine can also be converted to pyruvate, which can enter anaerobic glycolysis, which may indicate why this amino acid was decreased among EM patients in most of the reviewed studies [30]. Tryptophan may be fed into the kynurenine pathway to generate NAD+ for energy production in glycolysis [29]. The need for branched-chain amino acids, such as leucine, valine, and isoleucine, for tissue growth is similar to malignant conditions such as cancer [40]. The large need for branched-chain amino acids for cellular activities in endometriosis conditions may explain the alterations across multiple studies. Tissue growth also leads to a high turnover rate of structural protein, which could explain the increase in levels of amino acids such as arginine, tyrosine, leucine, lysine, and asparagine [37]. An increase in proline may be associated with fibrosis because this amino acid is involved in collagen synthesis [28]. One study noted that endometriosis is characterized by higher concentrations of reactive oxidative stress and lower glutathione levels [23]. A possible explanation for the alterations of histidine is to increase the synthesis of glutathione by increasing the levels of cysteine and glutamate [78]. Phenylalanine may be used in tissue repair, which could explain why it was reported to be decreased in endometrial tissue [36].

The reported changes in the lipidome of EM indicate vast changes to biochemical processes. The alteration of the lipidome may be related to damage induced by lipid peroxidation, altered cellular signals of apoptosis, and increased cell motility and proliferation [20,49,51]. Lipid peroxidation caused by reactive oxidative species may be a cause for the altered lipidome in EM [24]. Phosphatidylcholines can be converted into lysophosphatidic acids by phospholipase A2, and both compounds are thought to be involved in cell proliferation [50]. The increases in lysophosphatidic acid in one study [38] and the decrease in phosphatidylcholines [33,49] in two studies may be related to increased cell proliferation in EM. Sphingolipids and ceramides are involved in apoptosis, and sphingolipids also influence proliferation and motility, which are both factors in EM pathology [51]. Phosphatidic acids were noted to be increased in all the reported studies [38,48,49]. These are involved in inflammation, cell proliferation, phagocytosis, oncogenesis, and apoptosis [48]. The abundance of phosphatidic acids may contribute to the proliferative ability of EM. A sphingolipid, glucosylceramide, was found to be increased, and it is also associated with proliferative activities [43]. Some acylcarnitine metabolites, including lauroylcarnitine, hexadecenoylcarnitine, myristoylcarnitine, oleylcarnitine, and tetradecenoylcarnitine, were noted to be altered in the literature. Tetradecenoylcarnitine was elevated in both of the reported studies [39,45]. One article indicates that the presence of an increased acylcarnitine, C8:1, could be associated with inflammation [45]. The increase in the steroid estradiol metabolites found in serum may indicate that there may be an alteration of the aromatase enzyme [21]. Progesterone was hypothesized to suppress the COX-2 and HPGD expression in granulosa cells, which may be responsible for ovulatory dysfunction in EM [53].

Other compounds that were present in the metabolic signature of EM are noted to be associated with energy production and oxidative stress. Lactate was noted to be increased in the multiple studies [23,24,25,30,31,35,42]. An increase in this compound agrees with the earlier claim that EM is associated with increased anaerobic energy production. The increase in pyruvate may show that glycolysis may be enhanced in EM. The two TCA intermediates that were present in the results were citrate and succinate. Citrate was increased while succinate was decreased in the literature. This may indicate that there may be improper functioning of the TCA cycle. The build-up of succinate in EM may be a result of hypoactivity/hyperactivity of succinate dehydrogenase [26]. The binding of succinate to its receptor, SUCNR1, increases the inflammatory activity of macrophages, and it activates endometrial stromal cells, which play a role in forming lesions in EM [26]. Increases in ketone bodies as well as the decrease in glucose in some studies show that β-oxidation may be activated to produce more energy. The production of another compound, 3-hydroxybutyrate, is a ketone body that may be indicative of oxidative stress [29]. This compound was increased in all the studies that reported it as significant. An increase in 3-hydroxybutyrate may be associated with impaired bone marrow proliferation as well as lymphocyte proliferation [28]. These factors could influence the pathogenesis of EM. A similar compound, 2-hydroxybutyrate, can also represent the presence of reactive oxidative species [23].

Other EM-related compounds reported in the literature may be involved in the purine and pyrimidine metabolic pathways. Two purine metabolites, xanthine and hypoxanthine, were found to be increased in all the reported studies, which may indicate disrupted purine metabolism. Li et al. [37] hypothesized that purine nucleoside phosphorylase activity, which is involved in purine metabolism, may be reduced due to the build-up of purine metabolites such as inosine, hypoxanthine, xanthosine, and guanosine. Pyrimidine metabolites that were reported to be increased include deoxyuridine, thymine, and thymidine [20]. The increase in purine pyrimidine metabolites may potentially indicate DNA replication and ATP use [20]. One of the di-peptides, phenylalanyl-isoleucine, was reported to be increased in peritoneal endometriosis patients of all stages [39]. This compound is involved in intracellular transduction and possibly may increase growth in EM [39]. The increase in indole metabolites in the follicular fluid may be caused by tryptophan metabolism [20].

Overall, understanding the causes for the alterations in the metabolomic signature of EM helps provide insight into the pathogenesis as well as potential targets for treatment. As mentioned previously, there have been many discrepancies present in the research.

### 4.2. Limitations

The most notable limitation present in the literature is small sample sizes. The sample sizes in human models range from 16 to 180 total human subjects. Most human models contain a pool of subjects under 100. The present animal model studies also contain extremely low sample sizes, with the highest pool of samples in a case-control study being 46. Subtle differences in patient populations, such as age and BMI, may have amplified effects on the results of studies with smaller sample sizes. The low number of test subjects may skew results and could be a possible explanation for the large number of discrepancies found in the literature among the case-control studies.

Another limitation is the heterogeneity of the study populations and testing design. When comparing sample populations from different studies, there were many factors that made the populations different from one another. These factors include taking samples at different phases of the menstrual cycle, whether or not hormonal drugs were taken before the samples were taken, age, BMI, type of sample, phenotypes of EM, and stage of EM. One example is the discrepancy in lysine levels between studies. This discrepancy could be explained by something as simple as collecting samples at different stages of the menstrual cycle. The samples taken by Dutta et al. [36] were collected in the luteal phase, while the samples taken by Li et al. [37] were taken in the follicular phase. The differences in hormones of each menstrual cycle phase could have changed the metabolomic signatures of the tissue samples. Regarding the testing design, many of the studies used different identification techniques and methods for data analysis. The different study designs noted in this review introduce many variables and increase the difficulty in comparing studies and making accurate conclusions.

Multiple studies described in the present review omitted important information on the demographics of the sample population. Some did not factor in differences in ethnicity or lifestyle in their studies. Diet and differences in genetics could ultimately alter the metabolome and lead to altered results if not addressed. Although the stage of EM was reported in most of the studies, some studies also left out this important information. Lastly, some of the present studies did not present the individual phenotypes that make up the EM study group. Leaving out this vital information makes it challenging to understand the results of some studies.

In the present review, the potential for bias within the sources was not explicitly considered. No test was performed to assess the sources of internal bias, and this is a limitation. However, our aim was to be as inclusive as possible to provide a complete overview of all the metabolomic studies addressing EM. While we are confident that we have provided a thorough review of the body of EM metabolomics research, the subjective quality of the peer-reviewed studies was not addressed. The present systematic review was not registered a priori in the International Prospective Register of Systematic Reviews (PROSPERO) nor required to be registered [79].

### 4.3. Future Directions

Although it was difficult to obtain generalizable results about the metabolomic signature of EM, a few metabolite alterations were uniform between multiple studies. Specifically, 3-hydroxybutyrate, lactate, phosphatidic acids, succinate, pyruvate, tetradecenoylcarnitine, xanthine, hypoxanthine, L-isoleucine, and citrate were found to be consistently altered across multiple studies. These metabolites should be targets of future investigations. As the search continues for biomarkers or unique metabolomes of EM for identification, it may be beneficial to search for a panel of metabolites rather than focusing on a single biomarker. A study was able to distinguish EM from controls by using a panel of 20 metabolites with a sensitivity and specificity of 0.948 and 0.944, respectively [20]. Another approach is to investigate the ratio of metabolites. When the diagnostic potential of the proline to glutamine ratio in serum was investigated, a sensitivity of 74.29% and specificity of 85.42% was found [44]. Continuing to study notable metabolites and having multiple approaches for identification may help make the use of metabolomics in the non-invasive or minimally invasive identification of EM a more achievable task.

Future research in the field of metabolomics in EM needs to be conducted using larger sample sizes, as this will provide more reliable results on the metabolic fingerprint of EM. A recent review article that covered the topic of metabolomic biomarkers in EM also concluded that large-scale studies are required for the validation of biomarkers [16]. Although it is difficult to obtain large numbers of control and EM samples, such results may ultimately provide more uniform results. Animal case-control studies with larger sample sizes are also needed. As mentioned previously, metabolomics in animal models has shown similarities to those conducted in humans. It may be easier to obtain a larger sample size in animal models than in human models in certain cases.

Another important way to limit variation across the literature is by standardizing testing design. As mentioned in the Section 4.2 Limitations, there was a lot of variability across the identification techniques, data analysis, and sample populations. This factor makes it hard to compare the present research on biomarkers and may contribute to the large number of discrepancies in the current literature. Limiting variation among these factors through standardization of testing design will improve the reliability of research. Focusing future research on non-invasive sample sources may limit variability and also make obtaining larger sample sizes less challenging.

Future research must also account for all differences among sample populations. A review of the challenges of biomarker validation explains how minor changes in environmental factors such as diet, smoking, age, nutritional status, and even timing of collection can cause metabolite variation [80]. Since the metabolome is very sensitive, these factors must be addressed and documented in future research. Comparing different phenotypes of EM may also help improve disagreements and provide a new viewpoint on the pathology of EM. For example, the presence of an ovarian endometrioma in deep infiltrating EM was found to be associated with increased glycerol and ketone bodies in follicular fluid [30]. Another study found that ovarian endometrioma was also associated with higher ketone bodies and that deep infiltrating EM was associated with higher lipids in serum [22]. The differences in the signature of each phenotype show that these factors need to be addressed in future research. Other phenotypes of endometriosis, such as peritoneal lesions and infertility, may also need to be addressed in future research. Although the stage of EM was included in most studies, it is important that this information is documented.

In reality, the discovery of minimally invasive or non-invasive biomarkers appears to be a daunting task. For example, two studies were unable to distinguish an endometriosis cohort from a control cohort during multivariate and univariate analyses [81,82]. However, most of the present research has shown that the metabolomic profile of EM is significantly different from controls. Addressing the presented suggestions for future research may help lower differences in the outcomes of future metabolomics studies.

## 5. Conclusions

The pathogenesis of endometriosis is currently not well understood. Metabolomics has proved to be an effective way of studying biochemical changes associated with EM. Metabolomic studies using various sample types have shown that endometriosis can be distinguished from the control samples. These studies may be implemented in creating a non-invasive or minimally invasive test for the diagnosis of endometriosis. Animal models are an effective way of studying EM since they have shown similar results to those conducted in humans. Metabolomics can also give insight into the progression of symptoms, the correlation of external factors, and the treatment of EM. Although metabolomics has shown promising results, it must be noted that there are many discrepancies within the literature. Future research should include larger sample sizes, indicate differences in sample populations, such as phenotypes and stages of EM, and have a standardized testing design. These factors may help provide a more distinguishable metabolomic signature of EM and help reduce discrepancies in the future.

## Figures and Tables

**Table 1:** This table includes all of the significant metabolite differences among various sample types in human cohorts with and without EM. Arrows indicate that the metabolite was increased (up-arrow) or decreased (down-arrow) in patients with EM relative to those without EM. The studies are placed in columns that identify the sample source where the significantly altered metabolite was found. The stage of EM is noted after the source if it was presented in the article. Some of the most notable metabolites include 3-hydroxybutyrate, lactate, phosphatidic acids, succinate, pyruvate, tetradecenoylcarnitine, xanthine, hypoxanthine, L-isoleucine, and citrate.

Metabolite	Serum	Plasma	Follicular Fluid	Endometrial Fluid	Peritoneal Fluid	Urine	Tissue Sample	Cell Samples (Cumulus Cells and Granulosa Cells)
**1-methyladenosine**			↑ Wei et al. 2023 (III,IV) [20]					
**2-methoxyestradiol**	↑ Ghazi et al. 2015 (II,III) [21]							
**2-methoxyestrone**	↑ Ghazi et al. 2015 (II,III) [21]							
**2-octenoate**	↑ Maignien et al. 2020 (I–IV) [22]							
**2-hydroxybutyrate**	↑ Jana et al. 2013, ↑ Dutta et al. 2012 (I,II) [23,24]		↓ Castiglione-Morelli et al. 2019 (III,IV) [25]					
**2-hydroxyhippuric acid**					↑ Tian et al. 2024 (I–IV) [26]			
**2-hydroxy-3-methylpentanoic acid**					↑ Tian et al. 2024 (I–IV) [26]			
**2-hydroxyisovalerate**						↑ Vicente-Muñoz et al. 2015 (I–IV) [27]		
**3-hydroxybutyrate**	↑ Maignien et al. 2020 (I–IV), ↑ Dutta et al. 2012 (I,II), ↑ Angioni et al. 2023 (IV), ↑ Murgia et al. 2021 (IV) [22,24,28,29]		↑ Pocate-Cheriet et al. 2020 (IV) [30]					
**5-hydroxyindole acetic acid**			↑ Wei et al. 2023 (III,IV) [20]					
**5-hydroxymethyl-2-furancarboxylic acid**					↑ Tian et al. 2024 (I–IV) [26]			
**8-hydroxy-2′deoxyguanosine**			↑ Lazzarino et al. 2021 [31]					
**9,12,13-trihydroxy-10-octadecenoic acid**			↑ Wei et al. 2023 (III,IV) [20]					
**25-OH-cholecalciferol**			↓ Lazzarino et al. 2021 [31]					
**α Amino acids (not specified)**	↑ Braga et al. 2019 (III,IV) [32]							
**α-tocopherol**			↓ Lazzarino et al. 2021 [31]					
**β-pseudouridine**			↑ Lazzarino et al. 2021 [31]					
**β-D-glucose 6-phosphate**			↑ Wei et al. 2023 (III,IV) [20]					
**Acetoacetate**			↑ Pocate-Cheriet et al. 2020 (IV) [30]					
**Acetone**	↑ Maignien et al. 2020 (I–IV), ↓ Dutta et al. 2012 (I,II) [22,24]		↑ Pocate-Cheriet et al. 2020 (IV) [30]					
**Acetate**			↓ Castiglione-Morelli et al. 2019 (III,IV) [25]					
**Acylcarnitines**			↓ Wei et al. 2023 (III,IV) [20]		↓ Vouk et al. 2016 (III–IV), ↑ Li et al. 2021 [33,34]			
**Alanine**	↓ Jana et al. 2013, ↑ Dutta et al. 2012 (I,II), ↓ Angioni et al. 2023 (IV) [23,24,28]		↓ Pocate-Cheriet et al. 2020, ↓ Santonastaso et al. 2017 (I–IV) (IV) [30,35]				↓ Dutta et al. 2018 (I,II) [36]	
**Aldosterone**	↑ Ghazi et al. 2015 (II,III) [21]							
**Adipic Acid**	↑ Jana et al. 2013 [23]							
**All-trans-retinol (Vitamin A)**			↓ Lazzarino et al. 2021 [31]					
**Androstenedione**	↑ Ghazi et al. 2015 (II,III) [21]							
**Arachidonic acid**							↑ Li et al. 2018 (I,II) [37]	
**Ascorbic Acid**			↓ Lazzarino et al. 2021 [31]					
**Aspartate**			↓ Santonastaso et al. 2017 (I–IV) [35]					
**Bilirubin**			↓ Wei et al. 2023 (III,IV) [20]					
**Carnitine**			↑ Cordeiro et al. 2017 (III,IV) [38]		↓ Vouk et al. 2016 (III–IV) [33]			
**Cerimadine**					↑ Loy et al. 2021 (I–IV) [39]			
**Cholesterol**	↓ Ghazi et al. 2015 (II,III) [21]							
**Choline**			↓ Santonastaso et al. 2017 (I–IV) [35]					
**Citrate**			↓ Castiglione-Morelli et al. 2019 (III,IV), ↓ Pocate-Cheriet et al. 2020 (IV) [25,30]					
**Citric Acid**	↑ Jana et al. 2013 [23]							
**Creatine**	↓ Jana et al. 2013, ↑ Dutta et al. 2012 (I,II) [23,24]		↓ Pocate-Cheriet et al. 2020 (IV) [30]					
**Creatinine**		↓ Vicente-Muñoz et al. 2016 (I–IV) [40]				↑ Vicente-Muñoz et al. 2015 (I–IV) [27]		
**Cytosine**			↑ Lazzarino et al. 2021 [31]					
**Cytodine**			↑ Lazzarino et al. 2021 [31]					
**Dehydroepiandrosterone**	↑ Ghazi et al. 2015 (II,III) [21]							
**Deoxycorticosterone**	↑ Ghazi et al. 2015 (II,III) [21]							
**Deoxyuridine**			↑ Wei et al. 2023 (III,IV) [20]					
**Diacylglycerols**			↑ Cordeiro et al. 2017 (III,IV) [38]					
**Dihydroindole**			↑ Wei et al. 2023 (III,IV) [20]					
**Docosahexaenoic acid**								↑ Turathum et al. 2022 (CC) [41]
**Fatty Acids**	↓ Jana et al. 2013 [23]		↓ Santonastaso et al. 2017 (I–IV), ↑ Cordiero et al. 2017 (III,IV) [35,38]					
**Fucose**		↑ Vicente-Muñoz et al. 2016 (I–IV) [40]						
**Glucose**	↓ Jana et al. 2013, ↓ Dutta et al. 2012 (I,II) [23,24]		↑ Castiglione-Morelli et al. 2019 (III,IV), ↓ Pocate-Cheriet et al. 2020 (IV), ↓ Lazzarino et al. 2021, ↓ Santonastaso et al. 2017 (I–IV), ↑ Karaer et al. 2019 [25,30,31,35,42]					
**Glucose-1-phosphate**			↑ Wei et al. 2023 (III,IV) [20]					
**Glucosylceramide**	↑ Lee et al. 2014 (III,IV) [43]							
**Glutamine**	↓ Maignien et al. 2020 (I–IV), ↓ Kusum et al. 2022 (I–IV), ↑ Murgia et al. 2021 (IV) [22,29,44]		↑ Wei et al. 2023 (III,IV) [20]					
**Glutamic acid**	↓ Maignien et al. 2020 (I–IV) [22]							
**Glutamyl arginine**			↑ Wei et al. 2023 (III,IV) [20]					
**Glutamyl phenylalanine**			↑ Wei et al. 2023 (III,IV) [20]					
**Glutathione**			↓ Lazzarino et al. 2021 [31]					
**Guanosine**							↑ Li et al. 2018 (I,II) [37]	
**Guanidinosuccinate**						↑ Vicente-Muñoz et al. 2015 (I–IV) [27]		
**Glycerophophatidylcholine**	↑ Dutta et al. 2012 (I,II) [24]							
**Glycerophosphocholine**	↑ Jana et al. 2013 [23]							
**Hexadecenoylcarnitine**		↑ Letsiou et al. 2017 (III) [45]						
**Histidine**	↓ Maignien et al. 2020 (I–IV), ↑ Dutta et al. 2012 (I,II) [22,24]		↑ Wei et al. 2023 (III,IV) [20]					
**Hypoxanthine**			↑ Wei et al. 2023 (III,IV), ↑ Lazzarino et al. 2021 [20,31]				↑ Li et al. 2018 (I,II) [37]	
**Indole**			↑ Wei et al. 2023 (III,IV) [20]					
**Indole-3-acetamide**			↑ Wei et al. 2023 (III,IV) [20]					
**Indole Lactic acid**			↑ Wei et al. 2023 (III,IV) [20]					
**Inosine**							↑ Li et al. 2018 (I,II) [37]	
**Ketoleucine**			↓ Wei et al. 2023 (III,IV) [20]					
**Kynurenine**					↑ Li et al. 2021 [34]			
**Lactate**	↑ Jana et al. 2013, ↑ Dutta et al. 2012 (I,II) [23,24]		↑ Castiglione-Morelli et al. 2019 (III,IV), ↑ Pocate-Cheriet et al. 2020 (IV), ↑ Lazzarino et al. 2021, ↑ Santonastaso et al. 2017 (I–IV), ↑ Karaer et al. 2019 [25,30,31,35,42]					
**Lauroylcarnitine**		↑ Letsiou et al. 2017 (III) [45]						
**L-arginine**	↓ Jana et al. 2013, ↓ Dutta et al. 2012 (I,II) [23,24]	↑ Vicente-Muñoz et al. 2016 (I–IV) [40]	↓ Lazzarino et al. 2021 [31]				↑ Li et al. 2018 (I,II) [37]	
**L-asparagine**	↓ Jana et al. 2013 [23]						↑ Li et al. 2018 (I,II) [37]	
**L-isoleucine**	↓ Maignien et al. 2020 (I–IV), ↓ Jana et al. 2013, ↓ Dutta et al. 2012 (I,II) [22,23,24]		↓ Lazzarino et al. 2021 [31]					
**L-leucine**	↓ Maignien et al. 2020 (I–IV), ↓ Jana et al. 2013, ↑ Dutta et al. 2012 (I,II) [22,23,24]		↓ Lazzarino et al. 2021, ↓ Santonastaso et al. 2017 (I–IV) [31,35]				↓ Dutta et al. 2018 (I,II), ↑ Li et al. 2018 (I,II) [36,37]	
**L-lysine**	↑ Jana et al. 2013, ↑ Dutta et al. 2012 (I,II) [23,24]	↑ Vicente-Muñoz et al. 2016 (I–IV) [40]	↓ Santonastaso et al. 2017 (I–IV) [35]			↓ Vicente-Muñoz et al. 2015 (I–IV) [27]	↓ Dutta et al. 2018 (I,II), ↑ Li et al. 2018 (I,II) [36,37]	
**L-threonine**	↓ Maignien et al. 2020 (I–IV), ↑ Dutta et al. 2012 (I,II) [22,24]		↑ Pocate-Cheriet et al. 2020 (IV), ↓ Lazzarino et al. 2021 [30,31]					
**L-valine**	↓ Maignien et al. 2020 (I–IV), ↑ Dutta et al. 2012 (I,II), ↓ Angioni et al. 2023 (IV) [22,24,28]	↑ Vicente-Muñoz et al. 2016 (I–IV) [40]	↓ Castiglione-Morelli et al. 2019 (III,IV), ↓ Lazzarino et al. 2021, ↓ Santonastaso et al. 2017 (I–IV), ↑ Karaer et al. 2019 [25,31,35,42]			↑ Vicente-Muñoz et al. 2015 (I–IV) [27]		
**Lipids (not specified)**	↑ Jana et al. 2013, ↓ Dutta et al. 2012 (I,II) [23,24]		↑ Pocate-Cheriet et al. 2020 (IV), ↑ Santonastaso et al. 2017 (I–IV) [30,35]					
**Lysophosphatidic acids**			↓ Wei et al. 2023 (III,IV), ↑ Cordeiro et al. 2017 (III,IV) [20,38]					
**Lysophosphatidylcholines**			↓ Wei et al. 2023 (III,IV), ↑ Cordeiro et al. 2017 (III,IV), ↑ Sun et al. 2018 [20,38,46]		↑ Li et al. 2021 [34]			
**Lysophosphatidylethanolamine**							↑ Li et al. 2018 (I,II) [37]	
**Lysophosphatidylglycerol**			↑ Cordeiro et al. 2017 (III,IV) [38]					
**Lysophosphatidylinositol**			↓ Wei et al. 2023 (III,IV), ↑ Cordeiro et al. 2017 (III,IV), ↓ Dai et al. 2023 (I–IV) [20,38,47]					
**Myristoylcarnitine**		↑ Letsiou et al. 2017 (III) [45]						
**Malondialdehyde**			↑ Lazzarino et al. 2021 [31]					
**Methionine**			↓ Lazzarino et al. 2021 [31]					
**Monoacylglycerols**			↑ Cordeiro et al. 2017 (III,IV) [38]					
**N1-methyl-4-pyridone-5 carboxamide**						↑ Vicente-Muñoz et al. 2015 (I–IV) [27]		
**N-oleoylethanolamine**			↑ Wei et al. 2023 (III,IV) [20]					
**Nitrate**			↑ Lazzarino et al. 2021 [31]					
**Nitrite**			↑ Lazzarino et al. 2021 [31]					
**Oleamide**			↑ Wei et al. 2023 (III,IV) [20]					
**Oleylcarnitine**		↑ Letsiou et al. 2017 (III) [45]						
**Ornithine**	↑ Dutta et al. 2012 (I,II) [24]							
**Palmitic acid**								↑ Turathum et al. 2022 (CC) [41]
**Phosphatidic acids**			↑ Cordeiro et al. 2017 (III,IV), ↑ Dabaja et al. 2022 [38,48]				↑ Li et al. 2018 (I,II) [49]	
**Phosphocholine**			↓ Santonastaso et al. 2017 (I–IV) [35]					
**Phosphatidylinositol**			↑ Dai et al. 2023 (I–IV) [47]					
**Phosphatidylinositol bisphosphate**			↓ Cordeiro et al. 2015 (III,IV) [50]					
**Phosphatidylcholine**			↑ Wei et al. 2023 (III,IV), ↑↓ Cordeiro et al. 2015 (III,IV) [20,50]	↑↓ Domínguez et al. 2017 [51]	↓ Vouk et al. 2016 (III–IV), ↑ Loy et al. 2021 (I–IV) [33,39]		↓ Li et al. 2018 (I,II) [49]	
**Phosphatidylglycerol phosphate**			↓ Cordeiro et al. 2015 (III,IV) [50]					
**Phosphoethanolamine**					↑ Li et al. 2021 [34]			
**Phosphatidylserine**			↓ Cordeiro et al. 2017 (III,IV), ↑ Cordiero et al. 2015 (III,IV) [38,50]				↓ Li et al. 2018 (I,II) [49]	
**Phospholipids**			↑ Santonastaso et al. 2017 (I–IV) [35]					
**Phenylalanine**			↑ Wei et al. 2023 (III,IV) [20]				↓ Dutta et al. 2018 (I,II) [36]	
**Phenylalanyl-isoleucine**					↑ Loy et al. 2021 (I–IV) [39]			
**Plasmenylcholines**		↑ Vouk et al. 2012 (III,IV) [52]						
**Phytosphingosine**			↓ Sun et al. 2018 [46]					
**Primary bile acids**	↓ Ghazi et al. 2015 (II,III) [21]							
**Progesterone**	↑ Li et al. 2023 [53]		↑ Li et al. 2023 [53]		↑ Li et al. 2023 [53]			
**Proline**	↑ Angioni et al. 2023 (IV), ↑ Kusum et al. 2022 (I–IV) [28,44]		↑ Wei et al. 2023 (III,IV), ↓ Santonastaso et al. 2017 (I–IV) [20,35]				↑ Dutta et al. 2018 (I,II) [36]	
**Pyruvate**	↑ Jana et al. 2013 [23]		↑ Pocate-Cheriet et al. 2020 (IV), ↑ Karaer et al. 2019 [30,42]					
**Serine**			↓ Lazzarino et al. 2021 [31]					
**Succinate**	↑ Jana et al. 2013, ↑ Dutta et al. 2012 (I,II) [23,24]				↑ Tian et al. 2024 (I–IV) [26]			
**Sphingomyelins**		↑ Vouk et al. 2012 (III,IV) [52]		↓ Domínguez et al. 2017 [51]	↓ Vouk et al. 2016 (III–IV) [33]			↓ Turathum et al. 2022 (CC) [41]
**Sphingolipids**			↑ Cordeiro et al. 2015 (III,IV) [50]					
**Sphingosine**								↓ Turatham et al. 2022 (MGC) [41]
**Taurine**						↑ Vicente-Muñoz et al. 2015 (I–IV) [27]		
**Tetradecenoylcarnitine**		↑ Letsiou et al. 2017 (III) [45]			↑ Loy et al. 2021 (I–IV) [39]			
**Tetracosahexaenoic acid**					↑ Loy et al. 2021 (I–IV) [39]			
**Threonic acid**	↑ Angioni et al. 2023 (IV) [28]							
**Thymidine**			↑ Wei et al. 2023 (III,IV) [20]					
**Thymine**			↑ Wei et al. 2023 (III,IV) [20]					
**Trimethylamine-N-oxide**		↑ Letsiou et al. 2017 (III) [45]						
**Triacylglycerols**	↑ Braga et al. 2019 (III,IV) [32]			↓ Domínguez et al. 2017 [51]				
**Tyrosine**	↓ Maignien et al. 2020 (I–IV) [22]		↓ Pocate-Cheriet et al. 2020 (IV) [30]				↑ Li et al. 2018 (I,II) [37]	
**Tryptophan**	↓ Murgia et al. 2021 (IV) [29]		↑ Wei et al. 2023 (III,IV), ↓ Lazzarino et al. 2021 [20,31]					
**Unsaturated lipids**			↑ Castiglione-Morelli et al. 2019 (III,IV) [25]					
**Uracil**			↑ Lazzarino et al. 2021 [31]					
**Uric acid**							↓ Li et al. 2018 (I,II) [37]	
**Xanthosine**							↑ Li et al. 2018 (I,II) [37]	
**Xanthine**			↑ Wei et al. 2023 (III,IV), ↑ Lazzarino et al. 2021 [20,31]					

**Table 2:** Significant metabolites that differed between EM case and control animal models. The changes in metabolite levels are noted in EM models relative to the control group. Arrows indicate that the metabolite was increased (up-arrow) or decreased (down-arrow) in patients with EM relative to those without EM. The animal is noted beside the reference as well as the stage of EM if it was applicable. The two types of animals represented in the table are mice and non-human primates (NHP).

Metabolite	Serum	Tissue Sample	Peritoneal Lavage Fluid	Colonic Flushes	Feces
**2-aminoheptanoic acid**					↑ Chadchan et al. 2023 (Mouse) [54]
**2-Phosphoglycerate**	↑ Alghetaa et al. 2023 (Mouse) [55]				
**3-Phosphoglycerate**	↑ Alghetaa et al. 2023 (Mouse) [55]				
**12,13s-epoxy-9z,11,15z-octadecatrienoic acid**					↓ Ni et al. 2020 (Mouse) [56]
**Acetic Acid**				↓ Alghetaa et al. 2023 (Mouse) [55]	
**Alpha-linolenic acid**					↓ Ni et al. 2020 (Mouse) [56]
**Butyric acid**				↓ Alghetaa et al. 2023 (Mouse) [55]	
**Carnitine**		↓ Atkins et al. 2019 (NHP- II,IV) [57]			
**Chenodeoxycholic acid**					↑ Ni et al. 2020 (Mouse) [56]
**Creatine Phosphate**		↓ Atkins et al. 2019 (NHP- II,IV) [57]			
**FAD**		↓ Atkins et al. 2019 (NHP- II,IV) [57]			
**Fumarate**	↑ Alghetaa et al. 2023 (Mouse) [55]				
**Fructose-6-phosphate**	↑ Alghetaa et al. 2023 (Mouse) [55]				
**Glucose-6-phosphate**	↑ Alghetaa et al. 2023 (Mouse) [55]				
**Glutamate**	↑ Alghetaa et al. 2023 (Mouse) [55]				
**iso-butyrate**					↓ Chadchan et al. 2021 (Mouse) [58]
**Lactic Acid**					↑ Chadchan et al. 2023 (Mouse) [54]
**Lysophosphatidylcholines**	↑ Dutta et al. 2016 (Mouse) [59]		↑ Li et al. 2021 (Mouse) [34]		
**Lysophosphatidylethanolamine**	↑ Dutta et al. 2016 (Mouse) [59]				
**Malic acid**		↓ Atkins et al. 2019 (NHP- II,IV) [57]			
**Malate**	↑ Alghetaa et al. 2023 (Mouse) [55]				
**Maltose**					↑ Chadchan et al. 2023 (Mouse) [54]
**n-Acetyl aspartic acid**					↑ Chadchan et al. 2023 (Mouse) [54]
**n-butyrate**					↓ Chadchan et al. 2021 (Mouse) [58]
**NADH**		↓ Atkins et al. 2019 (NHP- II,IV) [57]			
**Phosphatidylcholines**	↑ Dutta et al. 2016 (Mouse) [59]				
**Phosphatidylethanolamine**	↓ Dutta et al. 2016 (Mouse) [59]				
**Phosphoenolpyruvate**	↑ Alghetaa et al. 2023 (Mouse) [55]				
**Phosphoethanolamine**			↑ Li et al. 2021 (Mouse) [34]		
**Plasmeny-phosphatidylethanolamine**	↑ Dutta et al. 2016 (Mouse) [59]				
**Propionic acid**				↓ Alghetaa et al. 2023 (Mouse) [55]	
**Quinic acid**					↑ Chadchan et al. 2023 (Mouse) [54]
**Sphingomyelins**	↑ Dutta et al. 2016 (Mouse) [59]				
**Succinate**	↑ Alghetaa et al. 2023 (Mouse) [55]				
**Triacylglycerols**	↓ Dutta et al. 2016 (Mouse) [59]				
**Tryptophan**		↓ Atkins et al. 2019 (NHP- II,IV) [57]			
**Ursodeoxycholic acid**					↑ Ni et al. 2020 (Mouse) [56]
**Valeric acid**				↓ Alghetaa et al. 2023 (Mouse) [55]	
**Valerate**					↓ Chadchan et al. 2021 (Mouse) [58]

## Data Availability

No new data were created or analyzed in this study. Data sharing is not applicable to this article.
